# Analyzing risky behaviors among different minority and majority race in teenagers in the USA using latent classes

**DOI:** 10.3389/fnbeh.2023.1089434

**Published:** 2023-02-14

**Authors:** Zeeshan Aslam, Muhammad Asim, Iqra Javaid, Faisal Rasheed, Muhammad Naveed Akhter

**Affiliations:** ^1^Nishtar Institute of Dentistry (NID), Multan, Pakistan; ^2^Nishtar Medical University, Multan, Pakistan; ^3^Wah Medical College, WahCantt, Pakistan; ^4^CMH Lahore Medical and Dental College, Lahore, Pakistan

**Keywords:** adolescence, demographic, LCA, sexual behavior, YRBSS

## Abstract

**Objective**: This study is to ascertain any inconsistencies in the trend of co-occurrence by sex of teenage health risk behavior patterns such as smoking, behaviors contributing to deliberate and unintentional injuries, risky sexual behavior, and sedentary lifestyle.

**Methods**: The study’s purpose was accomplished using Youth Risk Behavior Surveillance System (YRBSS) 2013 data. A Latent Class Analysis (LCA) was conducted for the entire sample of teenagers as well as separately for each sex.

**Results**: In this subset of youths, marijuana use was acknowledged by more than half of them, and smoking cigarettes was far more likely. More than half of the individuals in this subset engaged in risky sexual practices, like not using a condom during their most recent encounter. Males were split into three categories based on their involvement in risky behavior, while females were split into four subgroups.

**Conclusion**: Regardless of gender, various risk behaviors among teenagers are connected. However, gender variations in the higher risk of particular trends, such as mood disorders and depression among females, underline the significance of creating treatments that take adolescent demographics into account.

## Introduction

Adolescence and young adults in the United States are typically deemed to be in good health according to traditional indicators, including mortality, chronic illness, and hospitalization rates (Mulye et al., 2009; Shaw et al., 2015). According to research on the health condition of teenagers in the United States, the primary health dangers to adolescents are based on their behavioral decisions (Oh et al., 2019, 2022). Teenagers who are involved in unsupervised sexual activities have an increased risk of contracting STIs (Sexually Transmitted Infections) like HIV (Human Immunodeficiency Virus) and becoming pregnant unintentionally (Hamilton et al., 2010; Cavazos-Rehg et al., 2013). Adolescent risk behavior includes smoking, drinking alcohol, using illegal drugs, and engaging in risky sexual behavior, which can have negative physiological condition effects like unwanted pregnancy and sexually transmitted illnesses. This class also includes activities that expose participants to aggression and unintended harm (Bozzini et al., 2021). The individual’s personal well-being as well as his or her interactions with loved ones may suffer major effects if a continuous trend of harmful activity is not identified early on and adequately managed, even when these actions are often inconsistent (Tsevat et al., 2017).

Understanding teenage health-risk behaviors is crucial for implementing policies and creating successful preventative initiatives. Adolescent fatalities are most frequently brought on by accidental accidents (48%) and substance abuse (cigarettes, liquor, and other drug use). Teenagers who engage in unsupervised sexual activity have an increased risk of contracting STIs like HIV and becoming pregnant unintentionally. According to recent statistics, children between the ages of 15 and 19 experience an estimated 329,772 births, 548,032 instances of chlamydia, gonorrhea, and syphilis, and 2,240 cases of HIV per year (Chesson et al., 2010). Infertility, ectopic pregnancy, premature births, inflammation of the pelvis, and fetal abnormalities are among the harmful health effects of STIs in adolescents. Unwanted adolescent pregnancies may not only exacerbate socioeconomic limitations such as lower socioeconomic position and educational achievement but also raise the likelihood of adverse baby and mother health outcomes.

According to research, teenagers who are overweight or obese are more vulnerable to engaging in dangerous activities than their peers who are of a healthy weight (Farhat et al., 2010). In the US (United States), 32% of children ages 2–19 are overweight or obese. Poor eating practices, unhealthful weight control, and physical inactivity are examples of harmful behaviors that are established throughout adolescence and frequently followed into adulthood. Early sexual activity, having several partners, and unprotected sexual contact are all linked to liquor consumption (Fergusson and Lynskey, 1996). For instance, concurrent marijuana or drug use has been linked to the initiation of smoking (Wills et al., 2004), and drug use raises the chance of contemplating suicide (Park et al., 2006). Drug use and delinquent behavior have been linked to an increased risk of depression (Costello et al., 2008). The six high-risk behaviors that are the main causes of illness and death among children and young people in the US have been identified by the CDC (Centers for Disease Control). Some of the more prevalent ones are smoking, liquor and other drug use, sexual practices that increase the risk of unwanted pregnancy and STIs, bad eating habits, and inactivity.

Cigarette usage is highlighted as a significant public health challenge, particularly cigarette smoking (Costello et al., 2008; Owen and Halford, 2007). Smoking products accelerate the deterioration of lung function in children and teenagers. Smoking is linked to slowed lung development, persistent coughing, and wheezing. Although variations depending on other demographics are unknown, research findings have provided some indication that the trends or dangerous behaviors differ by gender (Centers for Disease Control and Prevention, 2014). 9% of American students in grades 9 through 12 reported using smokeless cigarettes, according to the 2013 Youth Risk Behavior Surveillance System (YRBSS). Smokeless cigarette use is linked to a number of harmful health effects, such as gingival depression, nicotine obsession, and oral, laryngeal, and pharyngeal malignancies (MacArthur et al., 2012). Cigarette makers have launched a plethora of colored brands with tastes that resemble candies. In an effort to attract young people, cigarette companies have more recently launched a plethora of vibrant brands with candy-like flavors, as well as smokeless and spitless products. Adolescent usage of alcoholic drinks, marijuana, and other illegal substances has been linked to morbidity and death. These chemicals have been related to a number of negative social and economic outcomes, such as family breakdown, crime, school leavers, and joblessness (Olajide et al., 2022).

The majority of Hispanic students have consumed alcohol at some point in their lives. According to the CDC, 35% of high school students in the United States currently use alcohol. Females are more prone than males to have consumed booze at some point in their lives (68% vs. 64%). Other high-risk behaviors (HRBs) in teenagers have been linked to alcohol consumption, including risky sexual behaviors (Khan et al., 2012; de la Haye et al., 2014), mental health disorders such as melancholy and suicidal behavior (Strachman et al., 2009; Fisher et al., 2018), and behaviors that Bleakley et al. (2017) mention. Liquor abuse, for example, has been associated with depression, suicidal thoughts and attempts, risky sexual behavior, aggressive behavior, poor academic or professional performance, and unexpected damage. Cannabis usage among high school students varies by race and ethnicity, with black and Hispanic students using it more frequently than white students (Owen and Halford, 2007). Usage of cannabis has been linked to aggressive conduct, delinquency, sadness, and violent behavior.

The incidence of other illegal drug usage among teenagers across the nation is as follows: 5% of teenagers say they have ever used cocaine in any form (powder, crack), 7% have used hallucinogens, 9% have used inhalants, 7% have used ecstasy, 2% have used heroin, 3% have used methamphetamines, and 18% have taken a prescription. 7% of people report using hallucinogens. 18% of people have used prescription medications without a prescription from a doctor. In the United States, accidental accidents are a significant cause of illness and death in adolescents and young people. Teenagers are more likely to drink liquor while driving, smoking, and engaging in risky sexual behavior (Parks et al., 2014). According to the most current statistics, one in four high school students said they had engaged in at least one violent altercation in the previous year (Khan et al., 2012). According to research, drug abuse is linked to aggressive conduct and actions that might cause harm (Weden and Zabin, 2005).

Gender refers to the array of socially constructed roles and relationships, personality traits, attitudes, behaviors, values, and relative power and influences that society ascribes to the two sexes on a differential basis. Simply put, sex refers to biological differences, whereas gender refers to social differences (Tsevat et al., 2017). Gender analysis in health has primarily been undertaken by social scientists who have discovered that biological differences alone cannot adequately explain health behavior. Health outcomes also depend on social and economic factors that, in turn, are influenced by cultural and political conditions in society (Khan et al., 2012).

The aim purpose of this study is to ascertain any inconsistencies in the trend of co-occurrence among different minority and majority races by sex of teenage health risk behavior patterns such as smoking, behaviors contributing to deliberate and unintentional injuries, risky sexual behavior, and sedentary lifestyle.

## Methods

The protocol for the YRBS was approved by the Institutional Review Board of the CDC. In order to preserve students’ privacy, survey protocols were created to enable voluntary, anonymous participation. Local parental approval procedures were followed prior to survey administration. The self-administered survey was completed by students during one class hour, and they wrote their answers directly on a computer-scannable booklet. Depression, a factor that influences hazardous and health-risk behaviors, was the main outcome factor. The performance measurement factors are examined as categorical variables in particular: (1) consensual sex with four or more people; (2) consensual sex with at least one person within the previous 3 months; (3) use of condoms; and (4) prior consumption of alcohol or drug use.

The YRBSS question “Sexuality?” served as the basis for our demographic variable, which was self-reported and quantified as a binary variable in the Latent class analysis (LCA). The descriptive statistics also contained data on age, academic level, and race/ethnic group. The individuals’ self-reported ages at the time the YRBSS was administered were used to compute chronological age. Based on two questions in the YRBSS, are you Hispanic? The racial group was assessed as a self-reported minimal variable. “Yes/No” and “What race are you?” Asian; Black or African American; Native Hawaiian or another Pacific Islander; White, American Indian, or Alaska Native the survey question “At what academic level are you?” serves as a gauge of the respondents’ academic level at the time of the survey.

The YRBSS, a nationally representative sample of American teenagers in grades 9 through 12, provided the data for the study. The Centers for Disease Control and Prevention created YRBSS to track six types of high-risk behavior for health. Using cigarettes, liquor, or other drugs, engaging in sexual conduct, or making poor food choices fall under these categories. Asthma and obesity prevalence are also monitored by YRBSS. The YRBSS data is collected every other year, in odd-numbered years, and represents all high school students enrolled that year. To collect data, anonymous self-administered questionnaires are employed. An LCA was performed to look for potential sex-specific patterns of hazardous behavior. The latent class analysis employed a total of 40 manifest variables, or items, that covered several issue behavior categories. The goal was to include as many potential issue behaviors as feasible in this list, including risk-taking activities like chatting while operating a motor vehicle. Each record includes a weight factor based on student gender, racial group, and school grade to adjust for student non-response and oversampling of black and Hispanic children. The final overall weights are modified such that the total sample size equals the weighted count of students, and the weighted proportions of students in each grade correspond to the country’s demographic estimations for each survey year. The findings are based on weighted proportions of students in each grade and national population forecasts for each survey year. Students who declined to participate in the state surveys or the national YRBS were not replaced in the sampled courses, schools, or students. The response rate for schools was 77.3%, while the response rate for students was 87.8%, for a response rate of 67.6%. All variables in this study were dichotomized in accordance with the 2013 YRBSS Data User’s Manual (Youth Risk Behavior Survey, 2013). The percentage of students reporting participation or not engaging in a behavior was indicated by dichotomous variables. Based on a favorable reaction to engaging in a behavior once or more during the course of the previous 12 months or a recent month, a behavior was considered to be dangerous (Nylund et al., 2007).

**Figure 1:**
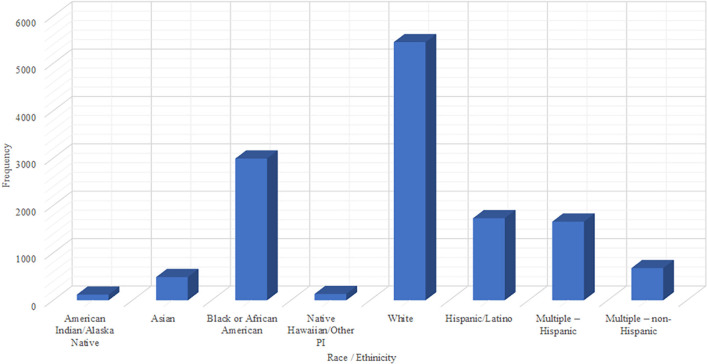
Bar chart showing Race Ethnicity of Youth Risk Behavior Surveillance System (YRBSS) 2013 survey.

**Figure 2:**
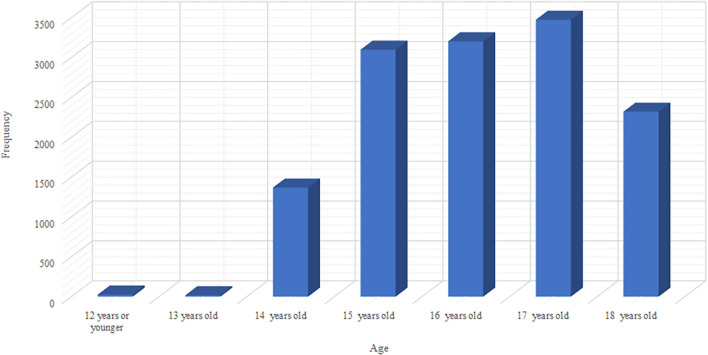
Bar chart showing the Age of YRBSS 2013 survey.

The survey comprised 13,583 teens from US high schools. Figure 1 show that more than half of the respondents were Caucasian (55.4%), and the majority were between the ages of 15 and 18 (Figure 2).

Latent class analysis (LCA) is a statistical technique used in this research to identify latent classes within a population based on their patterns of responses to multiple categorical or binary variables. The idea behind LCA is that individuals within a population may be grouped into different classes based on the similarities in their responses to multiple items or questions.

The basic assumptions of LCA is that the respondents in each class have a certain probability of giving a certain answer to each item and that these probabilities are class specific.

LCA can be considered a type of unsupervised machine learning algorithm, because it does not require prior knowledge about which individuals belong to which class, and the classes themselves are not directly observable. Instead, the classes are inferred from the patterns of responses to multiple items.

The likelihood ratio test (LRT) is a statistical test that compares a model with a more complex hypothesis to a model with a simpler hypothesis. The likelihood ratio test statistic (also known as the TECH 11 statistic) is a measure of the goodness of fit of a model and can be used to determine whether the more complex model provides a significantly better fit to the data than the simpler model.

### Statistical analysis

For analysis of the YRBSS data, SAS v. 9.4 was used to generate descriptive statistics so as to identify the socioeconomic and behavioral characteristics of the sample.

A structural main model was found by fitting an unconstrained latent class model to 40 risky behavioral signals. With respect to previous research, the model started with a two-class model and gradually added more classes till the best model class was matched with the data (Nylund et al., 2007). This strategy is used when there are no preconceived preconceptions about the grouping of manifest variables, as there were in this study (Chandler et al., 2021). The fitness was the ratio of probability, Akaike information criteria, revised Akaike information criterion, and the Bayesian information criterion. The latent class probabilities and conditional probabilities are derived using the population dispersion across latent classes, latent class characteristics, and entropy (Schreiber, 2022). Analyses were updated employing sample weights from the YRBSS to make the outcome relevant to high school students in the United States.

## Results

**Figure 3:**
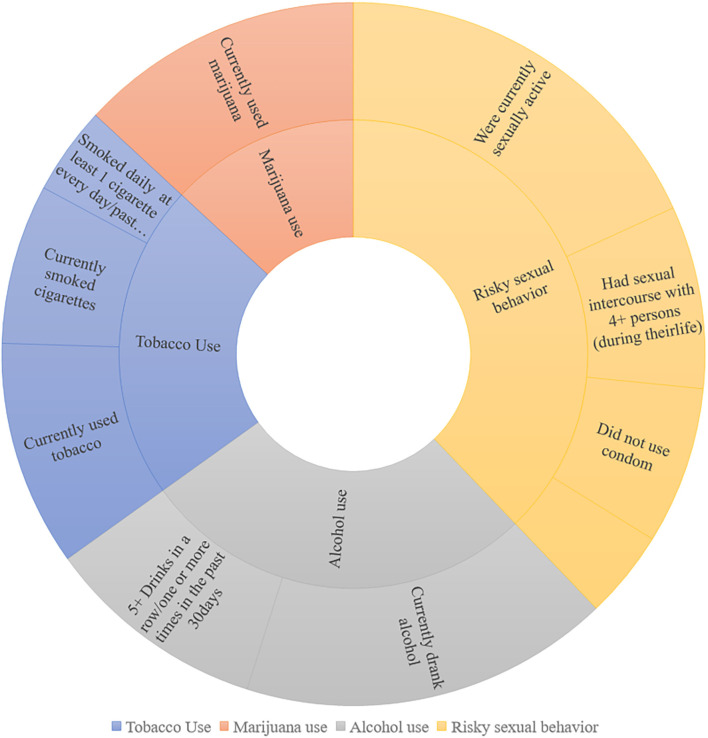
Sunburst of specific risk behaviors of YRBSS 2013 survey.

The prevalence of specific risk behaviors among the youth in our sample is shown in Figure 3. According to our research, many youths participate in risky behavior. Drug usage is common in this demographic, similar to reports by Thorsen (2018). Overall, 31.8% of young people had drunk alcohol on more than a day in the preceding month, and nearly a quarter had used marijuana on more than a day in the previous month. Almost 15.8% of teenagers reported being current smokers, defined as having smoked more than one cigarette in the preceding month. This generation also used prescription medications often, with around 18.3% of youngsters having done so without a doctor’s prescription in the preceding year. Teenagers in our sample demonstrated behaviors that resulted in unintentional harm and aggression, as seen in Figure 3. More than a quarter of the teenagers admitted to chatting while driving at least once during the month before.

A large number of students engage in risky sexual behavior. According to the findings, 32.3% of the young people in our survey are now interested in sex (i.e., had sexual relations with one or more people in the three months preceding the survey). 13.4% of sexually active teens at the time had not used a condom for their most recent experience.

The fitness for various classes taken into consideration in this study is shown in Table 1. From LCA, starting with a two-class solution and gradually adding more classes until the fit indices reached a plateau in terms of overall data fit for risk behaviors among teenagers in our sample, a five-class model gave the best results. The *p*-value for LRT, the value of entropy, and the review of BIC, were used to make this determination. On a scale of [0, 1], entropy is a measure of categorization precision, with values close to one denoting high classification confidence and values close to zero denoting poor classification confidence.

**Table 1:** Statistical description of health risk behaviors among adolescents.

Fit indices	2	3	4	5
AIC	367,453.6	376,590.6	373,116.9	373,426.2
ABIC	361,785.1	762,519.9	341,604.1	365,441.3
BIC	390,062.5	381,307.6	376,622.1	380,089.6
TECH 11 LRT	38,795.5	7,546.5	6,529.1	2,606.5
*p*-Value	0.00	0.35	0.47	0.63
Entropy	0.92	0.88	0.86	0.80

AIC, Akaike Information Criterion; BIC, Bayesian Information Criterion; ABIC, Sample size Adjusted BIC; TECH 11 LRT, The likelihood ratio test statistic; P-value, level of marginal significance; Entropy, measure of classification accuracy and defined on [0, 1].

Five types (subsets) of risky behavior were found in the sample of teenagers as a whole, as shown in Table 2. The majority of teenagers (52.6%) were in class 1. 14.9% of teenagers were in class 2. 14.3% of the teenagers were in class 3, 9.4% were in class 4, and 8.5% were in class 5, respectively. We included teenagers from Classes 1 and 2 who abstained from hazardous conduct. As a result, we classified the teenagers in these classrooms as low-risk. Compared to classes 1, 2, or 4, the adolescents in class 3 were more likely to be sad and suicidal. In one class, more than 79.8% of the teenagers reported having depression, 63.8% had experienced suicidal thoughts in the previous year, and more than 50.4% intended to commit suicide. Adolescents in class 4 who use cigarettes and liquor are shown. In comparison to the other classes, these teenagers had a higher likelihood of being current drinkers (more than one drink in the previous month) and current smokers (more than one cigar in the previous month). Class 5 was made up of high-risk youth who were more likely to engage in the most risky behaviors, including depression. These young people were classified as high-risk polydrug users. These teenagers had a high probability of taking prescription medications such as Codeine, OxyContin, Xanax, or Ritalin, in addition to a high probability of using nicotine, marijuana, and liquor. For the teenagers in this grouping, the likelihood of violent altercations over the previous 12 months was moderate.

**Table 2:** LCA of health risk behaviors among adolescents.

	Class 1	Class 2	Class 3	Class 4	Class 5
Currently Smoking Cigarette	0	0	0.004	0.97	0.972
Regular Cigarette Smoker	0.003	0.0029	0.0027	0.354	0.543
Currently Using Marijuana	0.044	0.462	0.168	0.543	0.827
Use of alcohol recently	0.064	0.876	0.305	0.746	0.954
Cocaine	0.002	0.064	0.043	0.054	0.543
Had sexual relations with at least four people	0.043	0.261	0.076	0.262	0.611
Engaged in sexual activity	0.164	0.614	0.271	0.573	0.834
Didn’t use condom	0.327	0.382	0.457	0.384	0.571
Took drugs or alcohol prior to previous sexual encounter	0.005	0.251	0.072	0.205	0.572

LCA, Latent Class Analysis.

A 4-class model for females in our sample and a 3-class model for males based on fit statistics was identified. In both groups of teenagers, 50.3% of females and 62.3% of males were classified as low-risk, also known as class 1.

More than a quarter of the males in Class 2 were teenagers. Due to the significant likelihood that they now consume liquor, the kids in this category were labeled as “liquor drinkers”. Males who were in Class 3 made up 12% of the sample and were classified as “High-risk Polysubstance Users”. Compared to sets 1 and 2, all members of this class showed a greater likelihood of using cigarettes, liquor, binge drinking, and cigarettes. In addition, compared to classes 1 and 2, males in this category were more likely to have used prescription drugs at some point in their lives. In Class 3, around two-thirds of the males admitted to carrying a weapon in the previous month, such as a knife, rifle, or club. Males in this set also had a high likelihood of getting into a fight physically at least once in the previous year.

Over 50% of the females in our sample belonged to Class 1, which is a low-risk subset. 20% of ladies in Class 2 supported the use of alcoholic beverages. Approximately half of the females in this cohort reported excessive drinking (more than five drinks of liquor in a row, within a couple of hours, more than a day in the previous month). Class 3 consisted of slightly more than 19% of the females in our sample who were classified as “liquor drinkers.” More than half of the females in this set acknowledged creating a suicide plan in the preceding year, and the majority of them experienced depressive symptoms and suicidal tendencies. We found that the females in this category were suicidal and sad. Almost 12% of the females were in Class 4. Females in this category were classified as "high risk" because they supported the majority of the risk behaviors that were investigated in our study. 80.3% of females reported smoking cigarettes, 74.5% reported using marijuana, and 88.3% reported drinking alcohol, with 71.4% reporting binge drinking. Additionally, 68.3% of these females supported using drugs, particularly prescription drugs. This subset of females also expressed suicidal ideation and depressive symptoms. Also, 79.3% of the females in this subset claimed to be sexually active; of those, 61.4% admitted to not wearing a condom during their most recent sex, and 44.4% admitted to using liquor or drugs just before their most recent sex.

## Discussion

### Key findings

The LCA identifies five subsets of youth interacting in risky behavior for the entire sample of adolescents in this study.

Class 1 and 2 (low risk): those who do not engage in risky behavior.

Those in Class 3 have depressive symptoms and suicidal thoughts.

Class 4: Those who have a high likelihood of using cigarettes, liquor,

Those classified as polysubstance users fall into Class 5.

In keeping with previous research, we observed that the majority of high school students (62% of males and 50% of females) were in the low-risk category. Adolescents with the highest risk profiles made up the smallest fraction of the population (12.2% for males and 11.6% for females). We discovered that young teenagers in the greatest subsets were involved in numerous risk behaviors, including substance usage, unsafe sex, and behavior patterns that resulted in accidental injuries and violence, lending credence to past studies that connected two or three risk behaviors (Finch, 2015; Thorsen, 2018). Because males and females had similar risky behavior categories, there were gender disparities in the volume and types of hazardous behavior among adolescents overall.

According to gender studies, females were more likely than males to have depressive symptoms, suicidal thoughts, and suicidal plans, as well as to be sedentary. Males in the polysubstance use subset exhibited considerably lower rates of depressive symptoms and suicidal thoughts and attempts than did females, who reported higher rates of both of these risk behaviors along with the majority of other risk behaviors. This result deviates from other studies that identified a specific risk profile among males, including high rates of marijuana use and suicidal behavior (Finch, 2015; Cheng et al., 2016). In our analysis, there were no discernible variations in the prevalence of drug use between males and females. Males reported using weapons and engaging in physical altercations at greater rates than females, though.

These findings support the issue behavior hypothesis by showing that risk behaviors co-occur and may have significant effects on efforts at prevention, intervention, and health promotion. These behavior modification initiatives must specifically acknowledge that health risk behaviors in this demographic are interconnected rather than discrete, unconnected activities. The majority of currently available interventions or programs concentrate on one or two main correlates that are thought to cause or contribute to problem behavior in adolescents, and these programs frequently offer a straightforward or one-dimensional approach to addressing and preventing risk behavior in adolescents. The Scared Straight program, for instance, targets young people who are delinquent or at-risk of becoming delinquent and aims to prevent them from engaging in criminal activity in the future. It is based on the idea that young people need to have a better understanding of the negative effects of their behavior as well as a change in attitude. The program’s elements include visits to prisons and talks by offenders to provide children an understanding of the harsh reality of life behind bars and the repercussions of their aggressive or illegal behavior (Ball and Weisberg, 2014; Cheng et al., 2016). This program posits that a specific undesirable behavior is caused by a single determinant, and that youngsters just need to be “Scared Straight” by witnessing the consequences of such negative behavior. Despite research showing that people who engage in initiatives comparable to Mortified are more inclined to perpetrate felonies in the future (Cheng et al., 2016), the program is nevertheless extensively employed across the country due to public safety concerns.

Specifically, the data from our research suggests that marijuana use is common among this group, with more than half of the individuals acknowledging use. Similarly, the data suggest that smoking cigarettes is also common, with a high likelihood of occurrence. In addition, more than half of the individuals in this group engage in risky sexual practices, such as not using condoms during their most recent encounter.

The LCA results also suggest that there are differences in the patterns of risky behavior among males and females in this group. Specifically, the LCA identified three categories of males based on their involvement in risky behavior, while four subgroups of females were identified. This suggests that there may be gender-specific patterns of risky behavior in this group of youths. It’s also worth noting that the high levels of risky behavior among this group are concerning, and it would be valuable to conduct further research to understand the factors that contribute to these behaviors in this group of youths. Additionally, identifying these subgroups could inform the design and delivery of public health interventions to target these specific at-risk subgroups and help to reduce the prevalence of these risky behaviors among them.

## Conclusion

Using LCA to examine each risk activity as a different but linked area of potentially dangerous adolescent behavior allows researchers to evaluate the co-occurrence of risky behaviors. This method allowed us to incorporate sex as a covariate and evaluate how patterns of certain behaviors affect participation in other hazardous behaviors.

LCA has been used to examine each risk activity as a different but linked area of potentially dangerous adolescent behavior and has helped to evaluate the co-occurrence of risky behaviors.

This research has helped in the understanding of how different risky behaviors may be related to one another and how they may interact to influence overall risk-taking behavior. Additionally, by incorporating sex as a covariate, research has evaluated how patterns of certain behaviors are affected by the participant’s sex. This has provided insights into how gender may influence the relationship between different risky behaviors and how the patterns of co-occurrence may differ between males and females. The LCA has provided a more nuanced understanding of risk-taking behaviors by identifying latent classes within the larger population based on their patterns of risky behaviors. This research has helped identify complex patterns of co-occurrence among multiple variables.

Integrated risk behavior prevention models must not only give correct information and impart useful life lessons for handling stress and conflict but they also must be strategically given while taking demographic aspects into consideration and be scheduled appropriately for adolescent growth. Interventions may be more successful and have a greater impact if they take into consideration the psychological and environmental factors that influence adolescent decision-making. In research looking at the impact of physical exercise on youth smoking cessation, implementing a multi-behavior program that addressed both smoking and physical activity in high school teenagers was shown to be much more successful than a short intervention in research. High schools were randomly allocated to one of three research conditions by Horn and colleagues: brief intervention, Not on Cigarettes, a tried-and-true program for teen smoking cessation, which also contained a physical activity module. The outcomes demonstrated that including a physical exercise component in cigarettes was very effective in helping males quit smoking.

There is a need for further research on health risk behaviors that makes use of representative sample observational studies with situational features. Our findings imply that future research should concentrate on sex variations in youths’ risky health behaviors. In our study, females were more often and significantly associated with polysubstance use and depression. Drug usage and either a low or high frequency of multi-partner sexual activity were significantly more correlated in females. Females are less likely than males to be classed as having several partners or as having a high multi-partner sexual risk, but when they use various drugs often, their relative risk for engaging in these behaviors increases. It may be essential to consider how one’s social and physical circumstances may affect behavioral decision-making while developing effective prevention and intervention techniques, as well as legislation relevant to adolescent health.

## Data availability statement

The original contributions presented in the study are included in the article, further inquiries can be directed to the corresponding author.

## Author contributions

ZA and FR: main manuscript writing and draft. MA, IJ, and MNA: editing, proofreading. All authors contributed to the article and approved the submitted version.

## References

[B35] Ball W. Weisberg R. (2014). The new normal? Prosecutorial charging in california after public safety realignment. SSRN Electronic J. 10.2139/ssrn.2403040

[B23] Bleakley A. Ellithorpe M. E. Hennessy M. Jamieson P. E. Khurana A. Weitz I. (2017). Risky movies, risky behaviors and ethnic identity among Black adolescents. Soc. Sci. Med. 195, 131–137. 10.1016/j.socscimed.2017.10.024 29146067 PMC5763912

[B7] Bozzini A. B. Bauer A. Maruyama J. Simões R. Matijasevich A. (2021). Factors associated with risk behaviors in adolescence: a systematic review. Braz. J. Psychiatr. 43, 210–221. 10.1590/1516-4446-2019-0835 32756805 PMC8023154

[B5] Cavazos-Rehg P. A. Krauss M. J. Spitznagel E. L. Schootman M. Cottler L. B. Bierut L. J. (2013). Characteristics of sexually active teenage girls who would be pleased with becoming pregnant. Matern. Child Health J. 17, 470–476. 10.1007/s10995-012-1020-0 22527768 PMC3517783

[B16] Centers for Disease Control and Prevention (2014). Youth Risk Behavior Survey Data. Available online at: www.cdc.gov/yrbs.

[B29] Chandler L. Abdujawad A. W. Mitra S. Mceligot A. J. (2021). Marijuana use and high-risk health behaviors among diverse college students post- legalization of recreational marijuana use. Public Health Pract. (Oxf) 2:100195. 10.1016/j.puhip.2021.100195 34888536 PMC8654161

[B33] Cheng T. J. Johnston C. Kerr T. Nguyen P. Wood E. K. DeBeck K. K. (2016). Substance use patterns and unprotected sex among street-involved youth in a Canadian setting: a prospective cohort study. BMC Public Health 16:4. 10.1186/s12889-015-2627-z 26728877 PMC4700772

[B9] Chesson H. Sternberg M. Leichliter J. Aral S. (2010). The distribution of chlamydia, gonorrhoea and syphilis cases across states and counties in the USA, 2007. Sex. Transm. Infect. 86, 52–57. 10.1136/sti.2009.040873 21098057

[B14] Costello D. M. Swendsen J. Rose J. S. Dierker L. C. (2008). Risk and protective factors associated with trajectories of depressed mood from adolescence to early adulthood. J. Consult. Clin. Psychol. 76, 173–183. 10.1037/0022-006X.76.2.173 18377115 PMC2659847

[B19] de la Haye K. D’Amico E. J. Miles J. N. V. Ewing B. Tucker J. S. (2014). Covariance among multiple health risk behaviors in adolescents. PLoS One 9:e98141. 10.1371/journal.pone.0098141 24858838 PMC4032285

[B10] Farhat T. Iannotti R. J. Simons-Morton B. G. (2010). Overweight, obesity, youth and health-risk behaviors. Am. J. Prev. Med. 38, 258–267. 10.1016/j.amepre.2009.10.038 20171527 PMC2826832

[B11] Fergusson D. M. Lynskey M. T. (1996). Alcohol misuse and adolescent sexual behaviors and risk taking. Pediatrics 98, 91–96. 8668418

[B32] Finch H. (2015). A comparison of statistics for assessing model invariance in latent class analysis. Open J. Stat. 5, 191–210. 10.4236/ojs.2015.53022

[B22] Fisher J. A. McManus L. Cottingham M. D. Kalbaugh J. M. Wood M. M. Monahan T.. (2018). Healthy volunteers’ perceptions of risk in US Phase I clinical trials: a mixed-methods study. PLoS Med. 15:e1002698. 10.1371/journal.pmed.1002698 30457992 PMC6245523

[B6] Hamilton B. E. Martin J. A. Ventura S. J. (2010). Births: preliminary data for 2008. National Vital Stat. Rep. 58, 1–18. 24979973

[B20] Khan M. R. Berger A. T. Wells B. E. Cleland C. M. (2012). Longitudinal associations between adolescent alcohol use and adulthood sexual risk behavior and sexually transmitted infection in the United States: assessment of differences by race. Am. J. Public Health 102, 867–876. 10.2105/AJPH.2011.300373 22493999 PMC3483900

[B17] MacArthur G. J. Smith M. C. Melotti R. Heron J. Macleod J. Hickman M.. (2012). Patterns of alcohol use and multiple risk behaviour by gender during early and late adolescence: the ALSPAC cohort. J. Public Health (Oxf) 34, i20–i30. 10.1093/pubmed/fds006 22363027 PMC3284864

[B1] Mulye T. P. Park M. J. Nelson C. D. Adams S. H. Irwin C. E. Jr. Brindis C. D. (2009). Trends in adolescent and young adult health in the United States. J. Adolesc. Health 45, 8–24. 10.1016/j.jadohealth.2009.03.013 19541245

[B28] Nylund K. L. Asparouhov T. Muthén B. O. (2007). Deciding on the number of classes in latent class analysis and growth mixture modeling: a Monte Carlo simulation study. Struct. Equ. Modeling 14, 535–569. 10.1080/10705510701575396

[B4] Oh H. Banawa R. Zhou S. Devylder J. Koyanagi A. (2022). The mental and physical health correlates of psychotic experiences among US college students: findings from the Healthy Mind Study 2020. J. Am. Coll. Health 1–7. 10.1080/07448481.2022.2058879. [Online ahead of print]. 35427464

[B3] Oh H. Waldman K. Stickley A. Devylder J. Koyanagi A. (2019). Psychotic experiences and physical health conditions in the United States. Compr. Psychiatry 90, 1–6. 10.1016/j.comppsych.2018.12.007 30639892

[B18] Olajide D. Eberth B. Ludbrook A. (2022). Analysis of multiple health risky behaviours and associated disease outcomes using scottish linked hospitalisation data. Front. Public Health 10:847938. 10.3389/fpubh.2022.847938 35899156 PMC9310786

[B15] Owen N. Halford K. (2007). Psychology, public health and cigarette smoking. Aust. Psychol. 23, 137–152. 10.1080/00050068808255600

[B13] Park H. S. Schepp K. G. Jang E. H. Koo H. Y. (2006). Predictors of suicidal ideation among high school students by gender in South Korea. J. Sch. Health 76, 181–188. 10.1111/j.1746-1561.2006.00092.x 16635202

[B24] Parks S. E. Johnson L. L. McDaniel D. D. Gladden M. Centers for Disease Control and Prevention (CDC) (2014). Surveillance for violent deaths—national violent death reporting system, 16 states, 2010. MMWR Surveill. Summ. 63, 1–33. 10.1016/j.jadohealth.2009.03.025 24430165

[B30] Schreiber J. (2022). Latent class and latent class regression. Contemp. Res. Methods Pharm. Health Services 55, 541–552. 10.1016/B978-0-323-91888-6.00044-2

[B2] Shaw P. H. Reed D. R. Yeager N. Zebrack B. Castellino S. M. Bleyer A. (2015). Adolescent and young adult (AYA) oncology in the United States: a specialty in its late adolescence. J. Pediatr. Hematol. Oncol. 37, 161–169. 10.1097/MPH.0000000000000318 25757020

[B21] Strachman A. Impett E. A. Henson J. M. Pentz M. A. (2009). Early adolescent alcohol use and sexual experience by emerging adulthood: a 10-year longitudinal investigation. J. Adolesc. Health 45, 478–482. 10.1016/j.jadohealth.2009.03.025 19837354 PMC2764540

[B31] Thorsen M. L. (2018). A latent class analysis of behavioral and psychosocial dimensions of adolescent sexuality: exploring race differences. J. Sex Res. 55, 45–59. 10.1080/00224499.2016.1254143 27982710 PMC5524591

[B8] Tsevat D. G. Wiesenfeld H. C. Parks C. Peipert J. F. (2017). Sexually transmitted diseases and infertility. Am. J. Obstet. Gynecol. 216, 1–9. 10.1016/j.ajog.2016.08.008 28007229 PMC5193130

[B26] Weden M. M. Zabin L. S. (2005). Gender and ethnic differences in the co-occurrence of adolescent risk behaviors. Ethn. Health 10, 213–234. 10.1080/13557850500115744 16087454

[B12] Wills T. A. Resko J. A. Ainette M. G. Mendoza D. (2004). Smoking onset in adolescence: a person-centered analysis with time-varying predictors. Health Psychol. 23, 158–167. 10.1037/0278-6133.23.2.158 15008661

[B27] Youth Risk Behavior Survey (2013). Division of Adolescent and School Health, National Center for HIV/AIDS, Viral Hepatitis, STD, and TB Prevention. Available online at: www.cdc.gov/yrbs.

